# Investigation of the physical, chemical, and biological properties of the cockle shell-derived calcium silicate-based pulp capping material: a pilot study

**DOI:** 10.1038/s41405-024-00281-w

**Published:** 2024-12-17

**Authors:** Orana Amonchaiyapitak, Busayarat Santiwong, Thanakorn Wasanapiarnpong, Theerapat Chanamuangkon, Pairoj Linsuwanont

**Affiliations:** 1 Department of Pediatric Dentistry, Faculty of Dentistry, Chulalongkorn University, 34 Henri-Dunant Road, Wangmai, Pathumwan, Bangkok, 10330 Thailand; 2 Department of Materials of Science, Faculty of Science, Chulalongkorn University, 254 Phayathai Road, Wangmai, Pathumwan, Bangkok, 10330 Thailand; 3 Biomaterial Testing Center, Faculty of Dentistry, Chulalongkorn University, 34 Henri-Dunant Road, Wangmai, Pathumwan, Bangkok, 10330 Thailand; 4 Department of Operative Dentistry, Faculty of Dentistry, Chulalongkorn University, 34 Henri-Dunant Road, Wangmai, Pathumwan, Bangkok, 10330 Thailand

**Keywords:** Dental biomaterials, Calcium-based cement

## Abstract

**Introduction:**

Hard-setting calcium hydroxide-based materials, e.g., Dycal and Life, have been widely used for direct pulp capping. However, various studies have shown undesirable effects such as high solubility and unpredictable dentine bridge formation. Bioceramic, mainly composed of tricalcium and dicalcium silicates, e.g., mineral trioxide aggregate and Biodentine, have provided more desirable physical and biological properties. This study aims to measure the physical properties, chemical properties, and biological response of human dental pulp cells (HDPCs) on three dental pulp-capping materials, Dycal, Life, and cockle shell-derived tricalcium silicate pulp capping material (C-Cap).

**Methods:**

C-Cap was prepared from cockle shells and rice husk ash. Its chemical composition was identified using X-ray diffractometry. The setting time, flow, solubility, and radiopacity tests were performed following the International Organization for Standardization 6876:2012. pH and calcium ion release were measured. The materials were subjected to an extraction medium at various concentrations and subsequently measured for cytotoxicity and migration on HDPCs, from three healthy, mature permanent teeth from different donors. Osteogenic differentiation was assessed by examining alkaline phosphatase enzyme activity and alizarin red staining assay. The data were tested for a normal distribution. The differences among groups were statistically analyzed using ANOVA and Tukey’s multiple comparison test (*p* < 0.05).

**Results:**

The setting time of each material was approximately 1–2 min. C-Cap showed the lowest solubility (10.27% ± 1.02%) compared to Dycal (12.67% ± 0.94%) and Life (12.74% ± 1.33%), with a significant difference (*p* < 0.05). All materials exhibited radiopacity ranging from 2.4 to 2.9 mm of aluminum. C-Cap had the highest flow, alkalinity, and calcium ion release. C-Cap was significantly less cytotoxic than Dycal and Life (*p* < 0.05). The migration of HDPCs cultured in C-Cap extraction medium (27.74% ± 0.12%) was comparable to that in serum-free medium (27.09% ± 0.08%) with a significant difference (*p* < 0.05). The mineralization by HDPCs maintained in C-Cap extraction medium was significantly higher than those in Dycal and Life extraction mediums with a significant difference (*p* < 0.05).

**Conclusions:**

C-Cap, a tricalcium silicate-based pulp capping material has potential for further development. C-Cap exhibited comparable physical properties and superior biological properties when compared to Dycal and Life.

## Background

Direct pulp capping (DPC) is a vital pulp therapy treatment [1] for preserving pulp vitality. During DPC, the pulp capping material is placed on an exposed area to protect the dental pulp from microbial infection and other irritations and stimulate pulp healing by forming reparative dentin. Choosing the right pulp capping material is crucial for the success of DPC. The material must have properties that prevent infection, minimize inflammation, and promote osteogenic differentiation to ensure effective treatment outcomes [2].

Calcium hydroxide has been widely used for DPC due to its antimicrobial activity, biocompatibility, and ability to promote hard tissue mineralization [3, 4]. Hard-setting calcium hydroxide-based materials, such as Dycal and Life, have been extensively used because they are easy to handle and produce satisfactory healing outcomes [5]. Currently, bioceramics, mainly composed of dicalcium and tricalcium silicates, e.g., mineral trioxide aggregate (MTA) and Biodentine, have been widely used for vital pulp therapy. Previous studies revealed that MTA has a higher biocompatibility, and is more predictable in dentin bridge mineralization than calcium hydroxide [6–8]. A systematic review and meta-analysis also reported that MTA-treated teeth had a better clinical success rate than calcium hydroxide when used as a pulp capping material [9].

Calcium silicate-based powder can be produced from precipitated calcium carbonate (CaCO_3_) and silica dioxide (SiO_2_). However, mining limestone for calcium carbonate manufacture raises environmental concerns due to deterioration of water quality, degradation of landscapes, and air/noise pollution [10–12]. To decrease the environmental impact of mining, calcium carbonate can be derived from biological sources. Cockle shells, a food industry byproduct, are high in calcium carbonate, while silicon dioxide is obtained from rice husk ash [13, 14]. Cockle shell-derived calcium carbonate nanoparticles are known as a non-toxic delivery vehicle for bone-remodeling agents and are efficient as medical nanocomposite bone scaffolds [15, 16]. In dentistry, nano-particle calcite was used as a calcium source for enamel remineralisation and treating hypersensitivity [17]. With its biocompatibility and remineralisation properties, it can be used to manufacture a pulp capping material. However, the effect of the cockle shells derived calcium silicate-based material on HDPCs and its physical properties has not been reported.

This study aimed to investigate a novel Calcium silicate-based the cockle shells derived calcium silicate-based material’s physical properties and its biological response on HDPCs compared with Dycal and Life.

## Methods

### Material preparation

Three materials were tested in this study: Dycal® (Densply, USA), Life® (Kerr, USA), and the newly developed tricalcium silicate (C-Cap). Dycal® and Life® were prepared per the manufacturer’s instructions. C-Cap was prepared from cockle shells and rice husk ash, as raw materials of tricalcium silicate. 500 g of cockle shell was washed, crushed, and boiled in 5% acetic acid 1 liter for 1 h and then boiled with distilled water 1 liter for 1 h. 500 g of rice husk ash was washed, boiled in 5% acetic acid 1 liter for 1 h, and then boiled with distilled water 1 liter for 1 h. Cockle shell and rice husk ash were dried at 105 °C for 24 h. The mole ratio of cockle shells to rice husk ash was 3:1 following this reaction

$$3{{{{\rm{CaCO}}}}}_{3}+{{{{\rm{SiO}}}}}_{2}\to {{{{\rm{Ca}}}}}_{3}{{{{\rm{SiO}}}}}_{5}+{3{{{\rm{CO}}}}}_{2}$$

The mixture was dry ball milled into powder using a zirconia planetary mill (Pulverisette 6, Fritsch, Germany) at 400 rpm for 30 min. The powder was compacted into 3-cm diameter, 1 cm thick molds. The molds were inserted in an alumina crucible, heated to 1450 °C for 2 h, and then cooled with a fan. The heated materials were crushed in an alumina mortar and mixed with 20% wt. calcined zircon powder (Cernic International Co., Ltd.) The powder was wet ball milled with ethanol as a milling medium, using a zirconia planetary mill at 400 rpm for 30 min and then dried at 80 °C overnight. X-ray diffractometry (XRD) was used to identify the tricalcium silicate powder’s composition. The particle size was determined using a dynamic laser scattering particle size analyzer. 25% wt zirconium silicate and 1% wt amorphous silicon dioxide were added to the tricalcium silicate powder. Methyl salicylate and N-Butyl benzene sulfonamide were component of liquid part. When used, Powder and liquid were placed into a capsule and mixed for 8 s at 4000 vibrations per minute by an amalgamator. The powder: liquid ratio was 2.5:1.

Five specimens of each material were prepared and used in the following tests.

### Setting time measurement

The setting time was measured per ISO 6876:2012. Each material was mixed and placed into a 2 mm high and 10 mm diameter stainless-steel ring mold on glass plate. Then sample was placed into cabinet (37 ± 1 °C and 95% humidity). A 2 ± 0.1 mm diameter Gilmore indenter weighing 100 ± 0.5 g was placed on the horizontal surface of the sample 5 s interval. If there was an indentation, the needle was raised, the tip was cleaned and relocated to other spots. The time that identer failed to identify was recorded as the setting time.

### Flow measurement

A 0.05 ml sample of each material was placed at the center of a glass plate with 1 ml. tuberculin syringe. A second glass plate was placed on top, with an additional mass on the plate, totaling 120 g of load for 10 min. The flow value was determined by measuring the diameter of the compressed sample.

### Solubility measurement

Solubility was measured based on ISO 6876:2012. The samples were mixed and placed into a 2 mm high and 10 mm diameter stainless-steel ring mold on glass plate. Stored in cabinet (37 ± 1 °C and 95% humidity) for 24 hours. Two samples were weighed (x) with a digital balance, immersed in 50 ml deionised water in beaker A, and incubated at 37 °C and 95% humidity for 24 h. Beaker B was weighed (y) before the samples and water from beaker A was poured through a funnel-fluted filter into beaker B, followed by water evaporation at 110 °C and weighing (z). The solubility percentage was calculated by dividing the dissolved sample mass by the initial sample mass.

$$\% {solubility}=\frac{z-y}{x}\times 100$$

### Radiopacity measurement

The materials were mixed and placed in 1 mm thick, 10 mm diameter silicone molds. The samples were removed from mold and placed on intraoral size 4 image plates (Carestream CS 7600, GA, USA), adjacent to a 20 mm wide, 50 mm long, and 0.5–9 mm thick aluminum step-wedge with 0.5 mm increments. The target-to-plate distance was 300 mm, using an X-mind® DC x-ray machine (Tuusula, Finland) at 60 kV, 8 mA, and 0.160 s. After digital film processing, Trophy DICOM 6.3.0.0 software (Carestream Dental, GA, USA) was employed to compare the samples’ radiopacity density with the aluminum step-wedge, recorded in millimeter-thickness.

### pH value and calcium ion measurement

Each material was placed in a 1.5 mm diameter and 10 mm long open-ended plastic tube. 10 specimens of each material, 5 for pH value and 5 for calcium ion measure. Each tube was immersed in a sealed container with 10 mL deionised water at 37 °C for 3 h, then transferred the specimen into a new container with 10 mL deionised water at 37 °C. Repeatedly measured calcium ion and pH values in 1, 3, 7, 14, and 28 days. Calcium ion release was measured by atomic absorption spectrophotometry (Model Agilent 280FS AA, Australia), and the pH value was measured by a pH meter (pH meter, PH550, Clean Leau, Taiwan). The pH meter was calibrated before measuring a new material.

### Cell culture

The HDPC isolation protocol was approved by the Human Research Ethics Committee (HREC-DCU 2022-064, No. 072/2022). Dental pulp tissues were collected from 3 healthy mature permanent teeth that were extracted for orthodontic reasons at the Oral Surgery Department, Chulalongkorn University. The extracted teeth were washed with phosphate-buffered saline (PBS). The pulps were excised, cut into 1 × 1 mm pieces, and placed in 35-mm culture dishes (Corning, New York, NY). The explants were maintained in complete medium (CM) comprising 1% Dulbecco’s Modified Eagle’s Medium (DMEM, Gibco, USA), 1% L-Glutamax (GlutaMAX-1, Gibco, USA) 100 unit/ml penicillin, 100 µg/ml streptomycin and 250 ng/ml amphotericin B (Antibiotic-Antimycotic, Gibco, USA), and 10% Fetal Bovine Serum (FBS, Gibco, USA). The cells were incubated at 37 °C in a humidified 5% CO_2_ atmosphere at 95% humidity. Cells from passages 3–5 were used for the experiments.

### Extraction medium preparation

To prepare the extraction medium, each material was mixed and shaped into cylindrical rubber molds (5 mm diameter and 2 mm height, with a 0.7065 cm² surface area). After setting for 24-h at room temperature, the materials were immersed in CM at 37 °C, 95% humidity, and 5% CO_2_ for 24 h. Following the ISO 10993 guidelines, the surface area-to-medium volume ratio was set at 1.271 cm²/mL [10] The extracted medium from each material was filtered with 0.22 µm sterile filters (Merck Millipore, Billerica, USA) and stored at 4 °C until use. Each experiment used the same batch of extraction medium.

### Cell viability assay

The [3-(4,5-Dimethylthiazol-2-yl)-2,5-Diphenyltetrazolium Bromide] (MTT) assay was performed using serially diluted extraction medium, with concentrations of 100, 50, 25, and 10% [18–20], each assessed in triplicate. HDPCs (1 × 10^5^ cells/mL) were seeded in 96-well plates in CM and cultured for 24 h. Subsequently, each material’s extraction medium was replaced. HDPCs in CM were used as a control. Cell viability was measured on days 1, 2, and 3 using an MTT assay by replacing the extraction medium with 1 mg/mL MTT (EMD Millipore, Germany), and cells were incubated at 37 °C in 95% humidity containing 5% CO_2_ for 4 h to allow formazan crystal formation. The formazan crystals were dissolved in a Dimethyl sulfoxide solution (Amresco, VWR International USA) and were measured by a UV-visible spectrophotometer microplate reader (Bio-Tek Epoch II, VT, USA) using a 570 nm wavelength. The percentage of cell viability in each incubation time point was calculated.

$$\% {{{\rm{Cell}}}}\; {{{\rm{viability}}}}=\frac{{{{\rm{absorbance}}}}\; {{{\rm{of}}}}\; {{{\rm{sample}}}}}{{{{\rm{absorbance}}}}\; {{{\rm{of}}}}\; {{{\rm{control}}}}}\times 100$$

### Migration assay

HDPC migration was investigated using a wound healing assay. HDPCs (5 ×10^4^ cells/ml) were seeded in 24-well plates in CM and incubated for 24 h, then the medium was replaced with serum-free medium for another 24 h to make a complete cell monolayer. A scratch wound was made by a cell scraper, and the CM and debris were removed. The extraction medium (25 and 100% of each material) was replaced, and CM served as a control. Microphotographs were taken at the initial time, 1 and 3 days at the exact location based on reference marks placed before the assay was performed. Images were captured using an inverted light microscope (Olympus, Japan) at a 4X magnification. The wound closure rate was determined using ImageJ (National Institute of Health, Bethesda, MD, USA). All samples were assayed in triplicate. The percentage migration was calculated.

$$	\% {migration}\\ 	 =\frac{{{{{\rm{area}}}}\; {{{\rm{of}}}}\; {{{\rm{wound}}}}\; {{{\rm{at}}}}\; {{{\rm{initial}}}}\; {{{\rm{time}}}}\; {-}\; {{{\rm{area}}}}\; {{{\rm{of}}}}\; {{{\rm{wound}}}}\; {{{\rm{at}}}}\; {{{\rm{observed}}}}\; {{{\rm{timepoint}}}}}}{{{{{\rm{AVG}}}}\; {{{\rm{area}}}}\; {{{\rm{of}}}}\; {{{\rm{wound}}}}\; {{{\rm{at}}}}\; {{{\rm{initial}}}}\; {{{\rm{time}}}}}}\times 100$$

### Osteogenic differentiation

HDPCs were seeded at a density of 5 ×10^4^ cells/ml in 24-well plates and were incubated at 37 °C in a 5% CO2 and 95% humidity. When HDPCs cultures became 90–95% confluence, culture medium was replaced with an osteogenic induction medium. The components of the osteogenic induction medium were complete culture medium, 50 ug/mL ascorbic acid, 10 mM β-glycerophosphate, 100 nM dexamethasone, and extraction medium from 25% and 100% concentration of each material. The osteogenic induction medium without extraction medium was used as a control. The osteogenic induction culture medium was changed every 48 h. The mineral deposition was analyzed using ALP and alizarin red S staining. All samples were performed in triplicate.

### Alkaline phosphatase (ALP) activity and Alizarin red S staining

ALP staining was performed on days 7 and 14 using the ab83369 Alkaline Phosphatase Assay kit (Abcam). Absorbance was measured using a spectrophotometer at 405 nm with a Microplate reader (Epoch2C, Bio-tek, USA). Alizarin red staining was performed on days 21 and 28 of cell culture. The HDPCs were fixed with formalin for 15 min, washed 3X with distilled water, and treated with Alizarin Red staining solution (Sigma-Aldrich, CA, USA). The cells were incubated in the dark for 30 min with gentle agitation and washed 3X with distilled water. The quantitative assessment of deposited Alizarin Red was performed by treating the samples with a 10% cetylpyridinium chloride solution for 15 min, followed by absorbance measurement at 540 nm wavelength. All samples were performed in triplicate.

### Statistical analyses

The data were tested for a normal distribution expressed as mean ± standard deviation (SD). Biological experiments were repeated from cells obtained from three different donors (*n* = 3) and performed triplicated. In each time point, since the normal distribution of the data was verified, the differences among groups were statistically analyzed using one-way ANOVA and Tukey’s multiple comparison test. The statistical analyses were performed using Prism 9 (GraphPad Software, CA, USA). A *p*-value of <0.05 was defined as significant.

## Results

### XRD

**Fig. 1:**
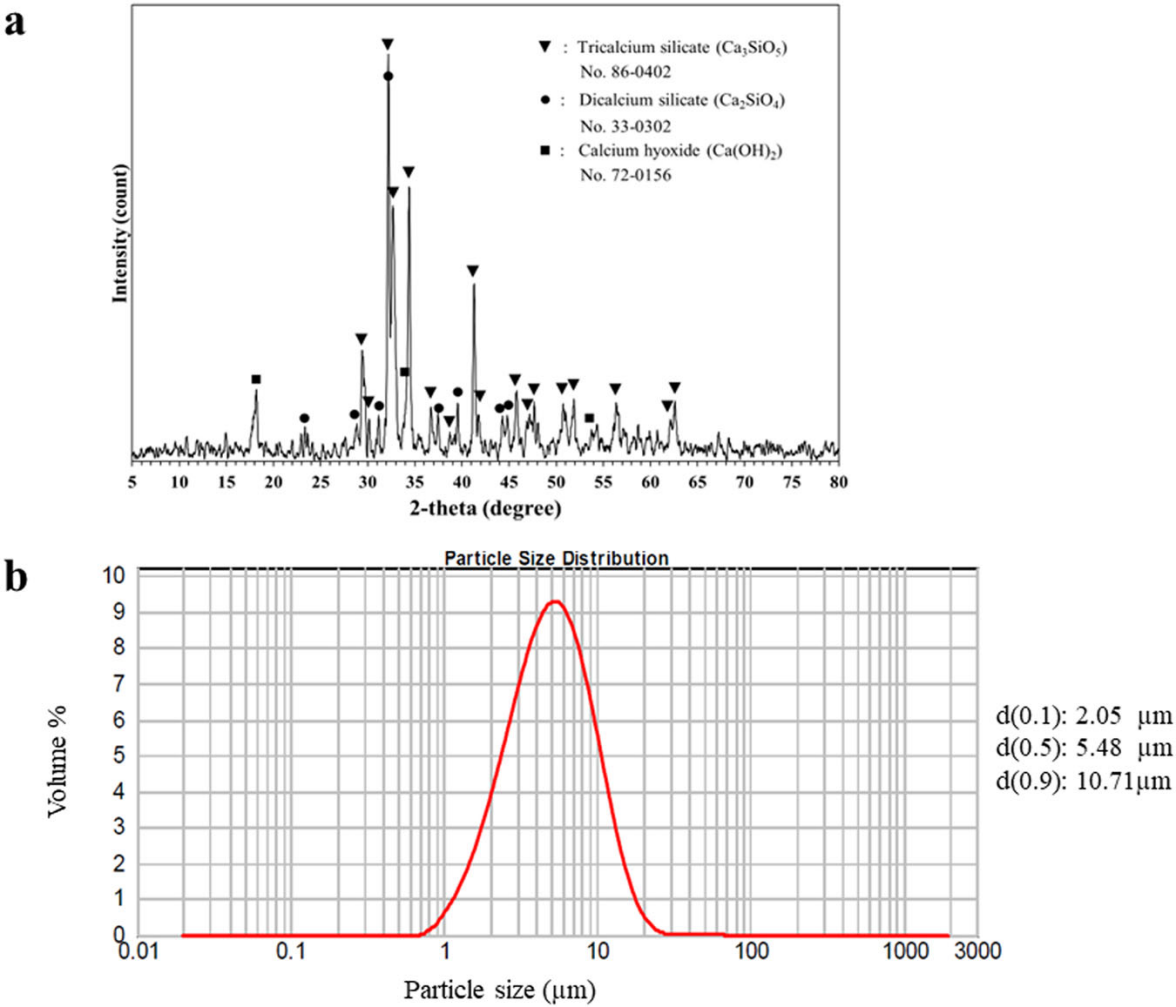
XRD analysis and particle size of the cockle shell-derived tricalcium silicate powder. **a** XRD analysis of the cockle shell-derived tricalcium silicate powder shows the peak of tricalcium silicate(▾), dicalcium silicate (●), and calcium hydroxide (■). **b** Particle size distribution of tricalcium silicate powder.

The XRD and quantitative phase analysis indicated that the major components of tricalcium silicate powder derived from cockle shells are tricalcium silicate (75%), dicalcium silicate (20%), and calcium hydroxide (5%) (Fig. 1a). The average particle size, according to the dynamic laser scattering particle size analyzer, was 5.48 microns (Fig. 1b).

### pH value and Calcium ion release

The pH value and Ca2^+^ release were determined (Table 1). At every time point, each material was alkaline. C-Cap had the strongest alkalinity among the materials, with a pH of 10.05–10.67. Life and Dycal® showed a pH range of 7.71–9.51. Each material comprised a calcium component and released calcium ions. The Ca^2+^ release from C-Cap was higher than from Dycal and Life at every observed time.

**Table 1:** pH value and calcium ion (ppm) of the tested materials at different time points (mean and standard deviation, *n* = 5).

Material		3 h	1 day	3 days	7 days	14 days	28 days
pH value	C	10.19 (0.09)^a^	10.06 (0.31)^a^	10.05 (0.59)^a^	10.65 (0.19)^a^	10.40 (0.75)^a^	10.67 (0.47)^a^
	D	9.51 (0.08)^b^	8.77 (0.11)^b^	9.02 (0.31)^b^	8.61 (0.42)^b^	8.22 (0.25)^b^	7.73 (0.30)^b^
	L	8.13 (0.35)^c^	8.56 (0.60)^b^	7.92 (0.16)^c^	7.71 (0.37)^c^	8.10 (0.03)^b^	9.26 (0.50)^c^
Ca^2+^ release	C	10.53 (0.74)^a^	17.79 (3.99)^a^	24.90 (1.10)^a^	23.77 (1.34)^a^	27.62 (10.38)^a^	42.35 (6.54)^a^
	D	3.59 (0.63)^b^	4.93 (0.74)^b^	8.30 (2.09)^b^	15.40 (10.16)^ab^	10.26 (2.70)^b^	11.69 (2.26)^b^
	L	4.92 (3.52)^b^	7.19 (4.71)^b^	12.49 (8.12)^b^	11.09 (8.04)^b^	16.21 (11.73)^ab^	13.80 (5.85)^b^

C-Cap (C), Dycal (D), and Life (L). Superscript lowercase letters indicate significant differences between materials (*p* < 0.05).

### Setting time, flow, solubility, and radiopacity

Every material had a setting time within the range of one to two minutes (Table 2). C-Cap showed the highest flow (*p* < 0.05). C-Cap also had the lowest radiopacity and solubility (*p* < 0.05).

**Table 2:** Setting time, flowability, solubility, and radiopacity of the tested materials (mean and standard deviation, *n* = 5).

Material	Setting time (Minute, second)	Flow (mm.)	Solubility (%)	Radiopacity (mm. of aluminum step wedge)
C-Cap	1, 20 (0, 04)^a^	25.59 (1.11)^a^	10.27 (1.02)^a^	2.494 (0.29)^a^
Dycal	1, 10 (0, 03)^a^	18.68 (0.09)^b^	12.67 (0.94)^b^	2.852 (0.08)^b^
Life	1, 53 (0, 13)^b^	22.82 (0.84)^c^	12.74 (1.33)^b^	2.572 (0.08)^ab^

Superscript lowercase letters indicate statistical differences between materials (*p* < 0.05).

### Cell viability

**Fig. 2:**
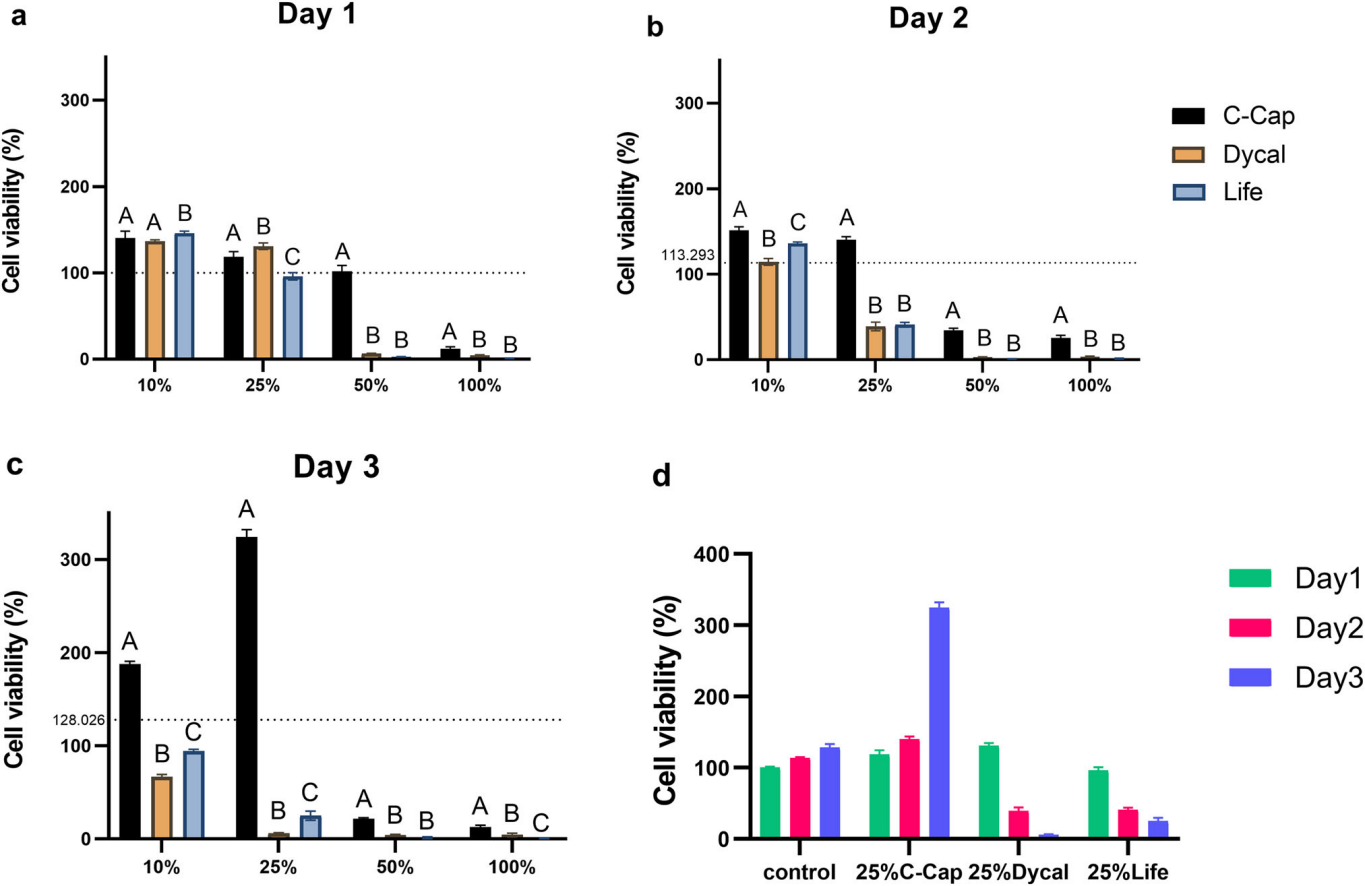
HDPC viability after being treated with 4 concentrations of extraction medium from C-Cap, Dycal, and Life was measured by MTT assay. The dashed line indicates the reference value of the CM used as control. **a** relative cell viability (%) 1 day after treated with each extraction medium. **b** 2 days, **c** 3 days. **d** evaluation of cell viability of HDPCs in complete medium and 25% extraction medium of each material at days 1–3. Different uppercase letters indicate statistical differences between each tested material only on the same day (ANOVA and Tukey’s multiple comparison tests, *p* < 0.05).

HDPCs were treated with 4 various concentrations of C-Cap, Dycal, and Life. Cell viability was determined by an MTT assay. After 1 day of treatment, the 100% concentration of each material exhibited higher cytotoxicity than the control (*p* < 0.05). Life reduced cell viability at 25%, 50%, and 100%, while Dycal and C-Cap reduced cell viability at 50% and 100%, respectively. C-Cap resulted in significantly higher viability than Dycal and Life at all concentrations (*p* < 0.05) (Fig. 2a).

On days 2 and 3, The cell viability of the C-Cap was significantly higher than Dycal and Life at equivalent concentrations (*p* < 0.05) (Fig. 2b, c). HDPCs treated with Life and Dycal exhibited decreased cell viability at all concentrations. C-Cap and control had increased cell viability from day 1 to day 3, while Dycal and Life had decreased cell viability on day 2 and day 3 (Fig. 2d). Moreover, HDPCs exposed to the various extraction media demonstrated a growth pattern that increased and decreased time- and concentration-dependent, respectively.

Based on the results of the cell viability assay, the cell migration assay, ALP activity, and Alizarin red test were measured using the 25% and 100% concentrations.

### Cell migration assay

**Fig. 3:**
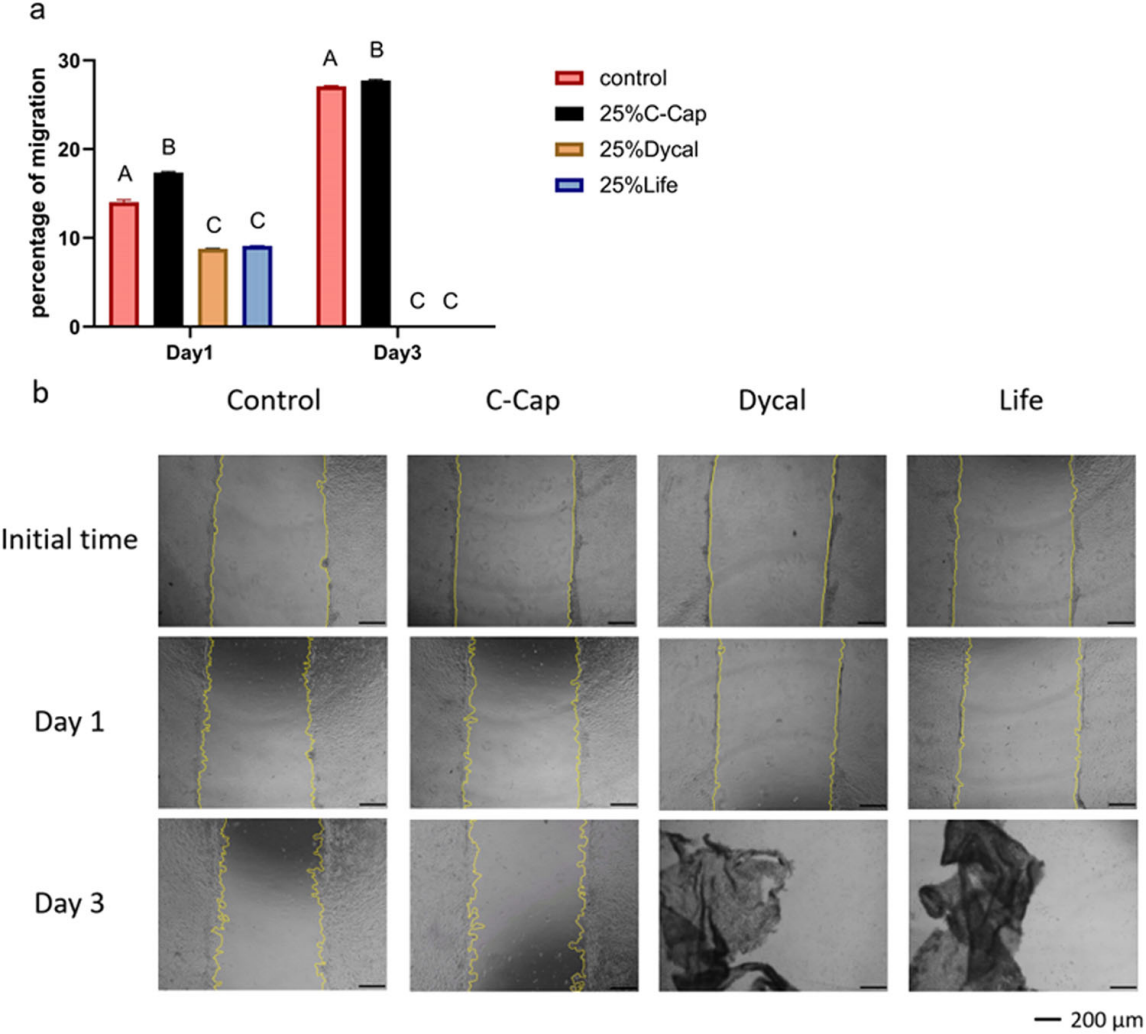
Cell migration was measured by scratch wound assay. HDPCs were treated with the 25% extraction medium of each material. The control was HDPCs in CM. **a** The percentage of cell migration at days 1 and 3 after the scratch was made. Different uppercase letters indicate statistical differences between each tested material only on the same day (ANOVA and Tukey’s multiple comparison tests, *p* < 0.05). **b** The images represent HDPCs before the scratch was made, the scratch area immediately after making the scratch, and 1, and 3 days after the test. The scratch areas were analyzed by ImageJ. The yellow lines indicate the open wound healing areas. Scale bar: 200 µm.

HDPC migration was assessed using an in vitro scratch wound assay, with evaluations conducted on days 1 and 3. C-Cap exhibited the highest percentage of cell migration, while Dycal and Life demonstrated lower migration rates that were not statistically different from one another on both days. By day 3, the wound area was reduced to 27.09% ± 0.08% in the control group and 27.74% ± 0.12% in the C-Cap group (Fig. 3a). Initially, the images displayed a distinct scratch wound border. By day 3, HDPC migration into the wound area was evident in both the C-Cap and control groups, while detached cells were noted in the Dycal and Life groups (Fig. 3b).

### Alkaline phosphatase (ALP) activity and Alizarin red S staining

**Fig. 4:**
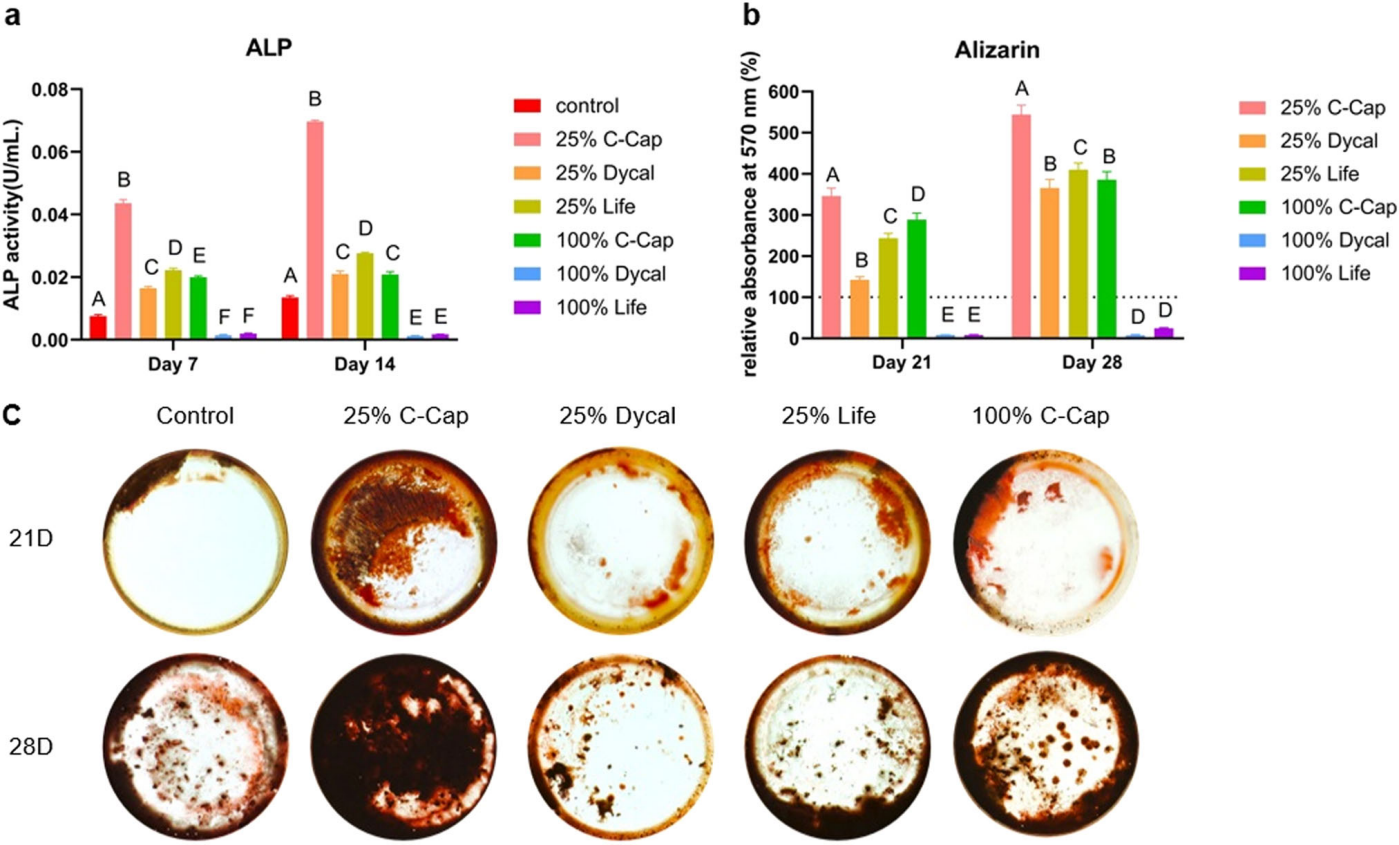
The effects of the extracted medium on HDPC osteogenic differentiation. **a** ALP activity after being treated with the extracted medium of each material at day 7 and day 14. **b** quantitative measurement of the relative absorbance of the eluted alizarin red S dye. The dashed line indicates the reference value of the control. Different uppercase letters indicate statistical differences between each tested material only on the same day (ANOVA and Tukey’s multiple comparison tests, *p* < 0.05). **c** Mineralized nodules formed by differentiated cells after incubation were stained with Alizarin Red S. HDPCs treated with OM were used as control.

HDPCs were treated with 25% and 100% of each extracted medium. OM served as the control group. ALP activity was measured on days 7 and 14 (Fig. 4a). The 25% C-Cap exhibited the significantly highest elevated ALP activity across all observed time points (*p* < 0.05). Regarding the 100% extracted medium, only C-Cap had higher ALP activity compared with control (*p* < 0.05), whereas 100% Dycal and Life exhibited lower ALP activity at every time point (*p* < 0.05).

HDPCs were stained with Alizarin red to assess mineral deposition on days 21 and 28. Figure 4b illustrates the relative absorbance at 540 nm, with the osteogenic medium (OM) used as a control (set at 100%). The greatest staining was observed for the 25% C-Cap group on both days. On days 21 and 28, all pairwise comparisons were significantly different, except for two comparisons: between 100% Dycal and 100% Life, and between 100% C-Cap and 25% Dycal. Images of the mineralized nodules indicated the highest level of mineralization in the 25% C-Cap group (Fig. 4c).

## Discussion

Calcium carbonate and silica dioxide are the raw ingredients to make tricalcium silicate [21]. Unlike traditional sources, e.g., Portland cement from limestone, this study produced tricalcium silicate using cockle shells and rice husk ash. Both materials, sourced from food and agricultural waste, are abundant, cost-effective, and environmentally sustainable.

Materials for clinical use should have desirable chemical, physical, and biological properties according to the ISO dental material testing guidelines. C-Cap demonstrated less solubility than Dycal and Life (10.27%, 12.67%, and 12.74% respectively), addressing a drawback of calcium hydroxide-based materials [22]. C-Cap has a creamy consistency that is easy to work with and a short setting time, which facilitates continuous restorative treatment. The C-Cap setting reaction is hydration [23], resulting in calcium hydroxide formation, which subsequently releases calcium ions and hydroxyl ions. High Ca^2^+ release and an alkaline pH suggest the potential for enhancing hard tissue mineralization and antibacterial effects [21]. All test materials exhibited a radiopacity value of less than 3 mm which is below the ISO criteria. For pulp liner material, radiopacity appearance may not be that important for clinical evaluation.

This study used the MTT assay, which is widely used to measure cell viability according to ISO10993-5;2009 [24]. To ensure comprehensive biocompatibility evaluation, multiple methods are used. Although typically employed for observing cell migration, the wound healing assay was adapted in this study to assess biocompatibility [25]. C-Cap had superior in vitro cell viability, while Dycal and Life exhibited cytotoxicity towards HDPCs. Our results correspond to a previous study, which reported better viability in a calcium-silicate based material, e.g., Biodentine [18].

Pulp capping materials have been documented to solubilize dentin, releasing growth factors that induce cell migration and promote pulp healing [26]. Early tissue healing involves recruiting progenitor cells to the injury site [27]. This study used a serum-free CM in the scratch wound assay to rule out the effects of serum proteins. Correlating the cell-cytotoxicity assays with the migration data, Dycal and Life showed a small area of migration on day 1 and cell detachment on day 3. C-Cap promoted significantly higher cell migration than control on day 1 and day 3. Reduced migration in the Dycal and Life groups might be related to their effect on cell viability. On day 3, Dycal and Life displayed cell detachment, potentially influenced by the cytotoxicity of the materials used. Additionally, the migration assay involved replacing the osteogenic medium with osteogenic medium (OM) every 24 h, which may have contributed to the detachment of dead cells. Correlated with the previous study, Dycal showed the highest percentage of dead cells, significantly inhibiting proliferation compared to other materials [28].

Mineralized tissue formation is the last key step after pulp-capping procedures [29]. The present study analyzed the mineralization effect of the materials based on ALP activity and Alizarin red assay. ALP is an early osteoblast differentiation indicator and is linked to mineralization. Elevated ALP levels indicate hard tissue formation [20]. HDPC hard tissue mineralization can be assessed by fluorescent calcein binding, Von Kossa staining, and alizarin red S staining [30, 31]. Alizarin red is a well-known dye for investigating biomineralization [32]. C-Cap demonstrated superior in vitro mineralization compared with control, Dycal, and Life. Similar ALP activity and Alizarin Red S results suggest that C-Cap induces mineralization in the early and late stages. The alizarin red analysis’s observed mineralization indicated only mineralized tissue formation. The osteogenic and dentinogenic markers analysis will elucidate the mechanism of mineralized tissue formation, which was not performed in this study. Similarly, Biodentine exhibited greater calcium deposition than Dycal in lipopolysaccharide-induced dental pulp stem cells using an Alizarin red assay [33]. Biodentine also resulted in increased mineralization in stem cells from the apical papilla and human mandible-derived mesenchymal stem cells [34, 35]. The tricalcium and dicalcium silicates in Biodentine were hypothesized as key factors for cellular responses [36], are also present in C-Cap. Due to the complex cell responses, in vivo studies are required to validate these pulp capping materials’ impact on tertiary dentin formations.

This study acknowledges several limitations. C-Cap was assessed solely against a widely used commercial calcium hydroxide-based pulp capping material. To provide a more comprehensive evaluation, future research should include comparisons with calcium silicate-based materials, such as mineral trioxide aggregate (MTA) or Biodentin. Additionally, subsequent investigations should incorporate in vivo studies using animal models and well-designed clinical trials to strengthen the validity and applicability of the findings.

## Conclusion

This pilot study demonstrated that C-Cap, a tricalcium silicate-based pulp capping material derived from food waste, has potential for further development. Despite the limitations of this study, C-Cap exhibited comparable physical properties and superior biological properties when compared to Dycal and Life.

## Data Availability

The datasets used during the current study are available from the corresponding author on reasonable request.
